# Inflammatory response biomarkers nomogram for predicting pneumonia in patients with spontaneous intracerebral hemorrhage

**DOI:** 10.3389/fneur.2022.1084616

**Published:** 2023-01-12

**Authors:** Tingting Yu, Haimei Liu, Ying Liu, Jianxin Jiang

**Affiliations:** ^1^Graduate School of Dalian Medical University, Dalian, China; ^2^Department of Neurology, The Affiliated Taizhou People's Hospital of Nanjing Medical University, Taizhou, China; ^3^Department of Neurology, Taizhou People's Hospital, Taizhou, China; ^4^Department of Neurosurgery, The Affiliated Taizhou People's Hospital of Nanjing Medical University, Taizhou, China; ^5^Department of Neurosurgery, Taizhou People's Hospital, Taizhou, China

**Keywords:** spontaneous intracerebral hemorrhage, pneumonia, nomogram, inflammatory response biomarkers, systemic inflammation response index, platelet/lymphocyte ratio

## Abstract

**Objectives:**

Inflammatory response biomarkers are promising prognostic factors to improve the prognosis of stroke-associated pneumonia (SAP) after ischemic stroke. This study aimed to investigate the prognostic significance of inflammatory response biomarkers on admission in SAP after spontaneous intracerebral hemorrhage (SICH) and establish a corresponding nomogram.

**Methods:**

The data of 378 patients with SICH receiving conservative treatment from January 2019 to December 2021 at Taizhou People's Hospital were selected. All eligible patients were randomized into the training (70%, 265) and validation cohorts (30%, 113). In the training cohort, multivariate logistic regression analysis was used to establish an optimal nomogram, including inflammatory response biomarkers and clinical risk factors. The area under the receiver operating characteristic (ROC) curve (AUC), calibration curve, and decision curve analysis (DCA) were used to evaluate the nomogram's discrimination, calibration, and performance, respectively. Moreover, this model was further validated in a validation cohort.

**Results:**

A logistic regression analysis showed that intraventricular hemorrhage (IVH), hypertension, dysphagia, Glasgow Coma Scale (GCS), National Institute of Health Stroke Scale (NIHSS), systemic inflammation response index (SIRI), and platelet/lymphocyte ratio (PLR) were correlated with SAP after SICH (*P* < 0.05). The nomogram was composed of all these statistically significant factors. The inflammatory marker-based nomogram showed strong prognostic power compared with the conventional factors, with an AUC of 0.886 (95% CI: 0.841–0.921) and 0.848 (95% CI: 0.799–0.899). The calibration curves demonstrated good homogeneity between the predicted risks and the observed outcomes. In addition, the model has a significant net benefit for SAP, according to DCA. Also, internal validation demonstrated the reliability of the prediction nomogram. The length of hospital stay was shorter in the non-SAP group than in the SAP group. At the 3-month follow-up, clinical outcomes were worse in the SAP group (*P* < 0.001).

**Conclusion:**

SIRI and PLR at admission can be utilized as prognostic inflammatory biomarkers in patients with SICH in the upper brain treated with SAP. A nomogram covering SIRI and PLR can more accurately predict SAP in patients' supratentorial SICH. SAP can influence the length of hospital stay and the clinical outcome.

## 1. Introduction

Spontaneous intracerebral hemorrhage (SICH) has high morbidity, mortality, and medical complications, in addition to primary brain injury, are significant causes of adverse outcomes (1, 2). After the development of stroke-associated pneumonia (SAP), unfavorable conditions may result in prolonged hospitalization, poor functional recovery, high social and economic burden, and even death (3). A study found that the median length of hospital stay was longer in patients with SAP (13 days) than in those without SAP (5 days) (4). Therefore, as a rapidly progressive disease with high mortality, early identification and effective indicators of SAP prevention are essential. Several risk factors for pneumonia in stroke patients, namely age, immunosuppression, dysphagia, previous medical history (i.e., diabetes, atrial fibrillation, alcohol consumption, and COPD), and stroke severity, were highlighted (5–8). However, these risk factors largely depend on clinical symptoms, and clinical monitoring of SAP remains imprecise. To predict SAP occurrence, an objective predictor is essential.

There is growing evidence that the immunodeficiency syndrome caused by stroke promotes the development of SAP, suggesting the significance of immune-inflammatory processes in SAP (9, 10). Routine blood markers (i.e., neutrophils, lymphocytes, and monocytes) are common systemic inflammation and infection markers. In addition, the systemic inflammation response index (SIRI), neutrophil/lymphocyte ratio (NLR), monocyte/lymphocyte ratio (MLR), and platelet/lymphocyte ratio (PLR) have better predictive power than conventional inflammatory factors (11–14). Most studies on risk factors for SAP are based on ischemic stroke. The pathophysiological mechanisms of SICH and acute ischemic stroke (AIS) are very different. Previous studies have shown that high NLR and SIRI predict SAP in patients with AIS (15, 16). However, the clinical significance of these inflammatory factors for SAP after SICH remains questionable. Furthermore, no predictive models of inflammatory indicators have been developed to predict the occurrence of SAP after SICH. Identifying risk factors is based on targeted primary prevention strategies and may influence clinical management by optimizing patient care.

This study established a predictive nomogram as a simple statistical visualization tool to predict disease onset, progression, prognosis, and survival (17–19). This study aimed to assess whether inflammatory response biomarkers on admission contribute to the early prediction of SAP after SICH.

## 2. Methods

### 2.1. Study population

**Figure 1:**
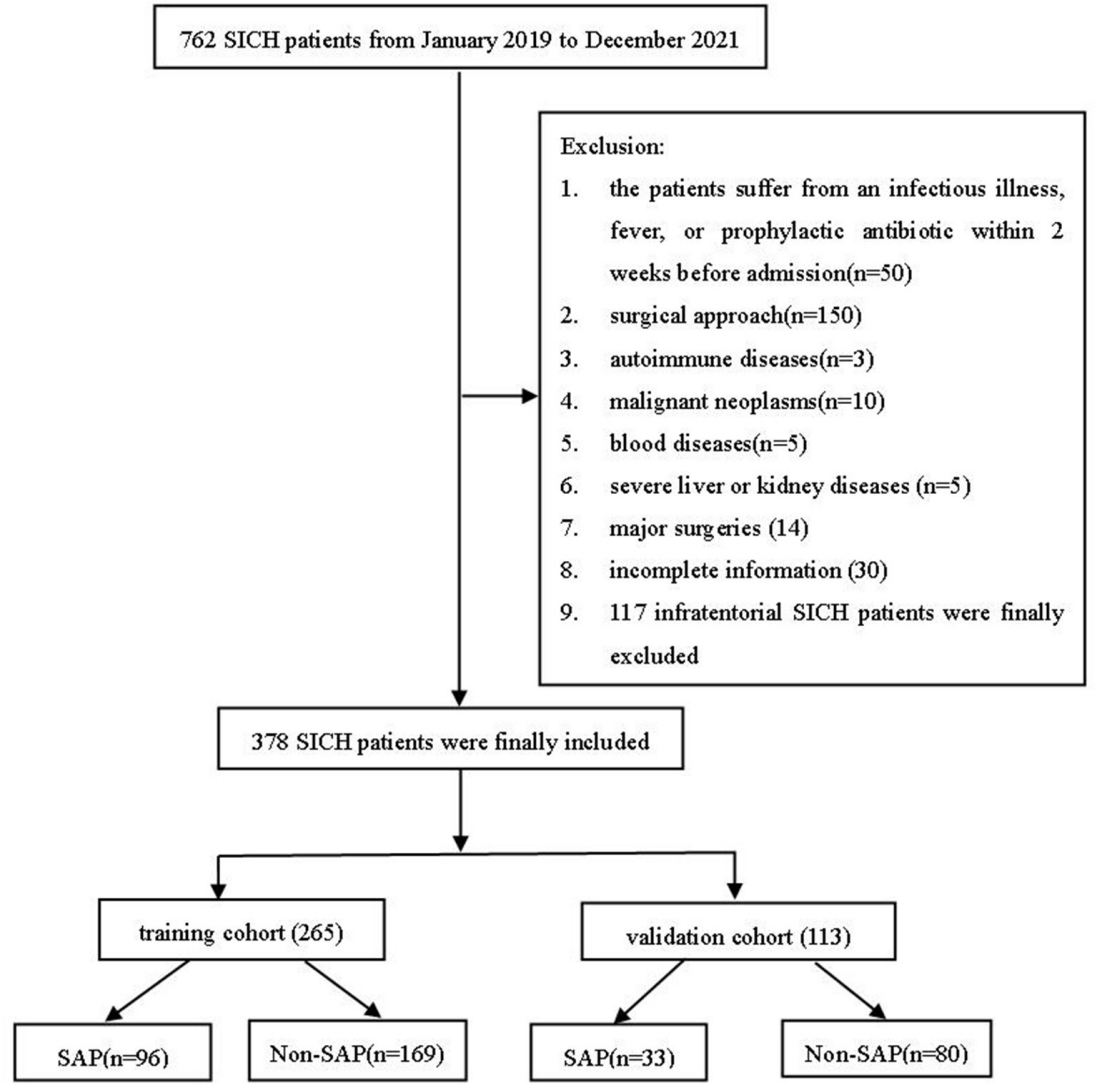
A flowchart of the present study. SICH, spontaneous intracerebral hemorrhage; SAP, stroke-associated pneumonia.

This observational study was approved by the local Ethics Committee of Taizhou People's Hospital and did not require individual patient consent (KY2022-094-01). The subjects of this retrospective study were patients with SICH who were admitted to Taizhou People's Hospital for conservative treatment from January 2019 to December 2021. SICH was determined by admission computed CT scanning. The decision of treatment modality of SICH (conservative treatment) was determined according to the diagnosis and treatment protocol, guidelines, and specific conditions of each patient. Inclusion criteria were: (1) CT diagnosis of SICH following the fourth national diagnostic criteria for cerebrovascular disease in 1995 in China, (2) CT follow-up within 24 h after admission, (3) age ≥18 years, (4) all patients were treated conservatively, and (5) diagnosing pneumonia was based on the diagnostic criteria for SAP in 2015 (20). We excluded patients with (1) infectious diseases, fever, or prophylactic antibiotics within 2 weeks before patient admission; (2) patients with infratentorial SICH; (3) cerebral hemorrhage due to trauma, subarachnoid hemorrhage due to aneurysm rupture and trauma; (4) patients after surgical treatment; (5) patients with autoimmune diseases, malignancies, hematological diseases, severe liver and kidney diseases, and history of major surgery; and (6) patients with incomplete information. We excluded patients with infratentorial cerebral hemorrhage. Patients with severe symptoms (GCS score ≤3) were excluded. Ultimately, 378 eligible patients were recruited. Patients were randomly classified into the training and validation cohorts in a 7:3 ratio (Figure 1).

### 2.2. Data collection

All participating patients were reviewed for a range of risk factors associated with SAP, including age, gender, body mass index (BMI), Glasgow Coma Scale (GCS), National Institute of Health Stroke Scale (NIHSS) score, systolic blood pressure (SBP) and diastolic blood pressure (DBP) at admission, time from onset to hospitalization, dysphagia, hematoma volume, hematoma location, and intraventricular hemorrhage (IVH). Medical history was collected, that is, hypertension, diabetes, atrial fibrillation, smoking, drinking, and antiplatelet or anticoagulation. Laboratory parameters (red blood cells (RBC), white blood cells (WBC), platelets, absolute neutrophil count (ANC), absolute monocyte count (AMC), absolute lymphocyte count (ALC), and albumin) were obtained within 24 h of admission for all subjects. We collected the length of hospital stay and the functional recovery of the patients. To obtain their functional recovery, we followed up the patients or their families 3 months after discharge using a telephone.

### 2.3. Measurements and study outcomes

A trained neurologist assessed the GCS and NIHSS scores at admission to assess the severity of SICH. The following formulae were used to compute the lymphocyte-based inflammatory index in this study: SIRI (21), NLR (15), MLR (14), and PLR (22) from the first peripheral blood count at admission.

The primary outcome of our study was SAP after SICH. Patients with signs or symptoms of respiratory infection underwent routine blood tests, and chest CT scans to diagnose pneumonia. Physicians from neurology and radiology jointly diagnosed pneumonia (20). Secondary outcomes were the length of stay and functional recovery. The modified Rankin Scale (mRS) score was utilized to evaluate functional recovery after 3 months, that is, an mRS score of 3–6 indicates poor clinical outcome.

### 2.4. Statistical analysis

Normally distributed continuous variables were expressed as mean ± standard deviation (M ± SD), and skewed distributions were expressed as median with interquartile range (IQR, Q1–Q3). Categorical variables are expressed as frequencies and percentages (%). Where appropriate, the *t*-test, the Mann–Whitney *U*-test, and the chi-square test were utilized for comparisons.

Multivariate logistic regression models considered variables with *P* < 0.05 in the univariate analysis results to obtain independent predictors. The Hosmer–Leeshawn test was utilized to assess the model's goodness of fit. In addition, a Nomogram with independent predictors was constructed from the training cohort. The area under the receiver operating characteristic (ROC) curve (AUC) and the calibration curves were utilized to assess the predictive power and compliance of the model. We performed a decision curve analysis (DCA) to quantify the net benefit of different threshold probabilities to determine the clinical utility of the nomogram we developed. After that, the visual prediction model was validated internally. Statistical analyses were performed on SPSS 26.0 (IBM Corporation, Chicago, IL) and R statistical software (R, version 4.1.1). Statistically significant differences were considered to be two-tailed at a *P*-value of < 0.05.

## 3. Results

### 3.1. Baseline characteristics

A total of 378 patients with SICH, including 265 in the training and 113 in the validation cohort, were included. Except for a statistically significant difference in GCS at admission (*P* < 0.05) regarding baseline characteristics, other variables did not differ between the two cohorts (Table 1). Patients with SAP (36. 2%) tended to be older, had higher rates of hypertension, antiplatelet or anticoagulation, dysphagia, and IVH, and had larger hematoma volumes, lower GCS scores, RBCs, ALC, hemoglobin, and albumin, and higher NIHSS scores, WBCs, ANC, SIRI, NLR, MLR, and PLR in the training cohort (*P* < 0.05, Table 2). Patients in the SAP group had a longer length of hospital stay (*P* < 0.05, Table 2). Three months after discharge, the mRS score of the SAP group differed from that of the non-SAP group (Table 2, *P* < 0.001). Clinical outcomes (mRS3-6) were significantly worse in the SAP group than in the non-SAP group (71.9 vs. 49.1%, *P* < 0.001, Table 2).

**Table 1:** Baseline characteristics of all patients in the training cohort and validation cohort.

	**Total** **(*n* = 378)**	**Training cohort** **(*n* = 265, 70.1%)**	**Validation cohort** **(*n* = 113, 29.9%)**	***P***
**Demographics**				
Age [year, M (Q1, Q3)]	63.54 ± 13.72	63.48 ± 13.75	63.67 ± 13.69	0.900
Gender, *n* (%)				0.572
Male	253 (66.9)	175 (66.0)	78 (69.0)	
Female	125 (33.1)	90 (34.0)	35 (31.0)	
BMI[kg/m^2^, M (Q1, Q3)]	24.49 (21.26, 27.08)	24.49 (21.48, 27.04)	24.22 (20.58, 27.31)	0.845
**Medical history**				
Hypertension, *n* (%)	283 (74.9)	198 (74.7)	85 (75.2)	0.918
Diabetes mellitus, *n* (%)	29 (7.7)	20 (7.5)	9 (8.0)	0.889
Atrial fibrillation, *n* (%)	26 (6.9)	19 (7.2)	7 (6.2)	0.732
Smoking, *n* (%)	97 (25.7)	71 (26.8)	26 (23.0)	0.441
Drinking (>3 drinks per 24 h), *n* (%)	86 (22.8)	57 (21.5)	29 (25.7)	0.378
Antiplatelet or anticoagulation, *n* (%)	26 (6.9)	18 (6.8)	8 (7.1)	0.920
**Clinical characteristics**				
NIHSS [score, M (Q1, Q3)]	3 (2, 8)	4 (2, 8)	3 (2, 6)	0.144
GCS [score, M (Q1, Q3)]	13 (10, 15)	13 (9, 15)	14 (12, 15)	0.002^#^
Admission SBP [mmHg, M (Q1, Q3)]	162 (150, 179.50)	164 (150, 181)	161.23 ± 20.17	0.106
Admission DBP [mmHg, M (Q1, Q3)]	95 (85, 105.5)	95.87 ± 16.12	94 (81, 105)	0.467
Duration from onset to hospitalization [h, M (Q1, Q3)]	5 (3, 12)	5 (3, 11)	5 (3, 12)	0.749
Dysphagia, *n* (%)	169 (44.7)	123 (46.4)	46 (40.7)	0.307
**ICH parameters**				
Hematoma volume[ml, M (Q1, Q3)]	12.16 (5.35, 23.34)	12.54 (5.38, 23.02)	12.00 (5.21, 24.65)	0.881
Hematoma location, *n* (%)				0.315
Lobar	82 (21.7)	52 (19.6)	30 (26.5)	
Basal ganglia region	225 (59.5)	161 (60.8)	64 (56.6)	
Thalamus	71 (18.8)	52 (19.6)	19 (16.8)	
IVH, *n* (%)	108 (28.6)	79 (29.8)	29 (25.7)	0.414
**Laboratory data**				
RBC [10^12^/L, (M ± SD)]	4.41 ± 0.62	4.37 (3.98, 4.82)	4.48 ± 0.62	0.195
Hemoglobin [g/L]	136 (122.5, 148)	135.03 ± 18.09	137 (122.25, 151.75)	0.541
WBC [10^9^/L, M (Q1, Q3)]	7.61 (6.08, 10.07)	7.63 (6.27, 9.80)	7.60 (5.92, 10.55)	0.884
ANC [10^9^/L, M (Q1, Q3)]	5.52 (4.14, 8.09)	5.53 (4.26, 8.03)	5.34 (4.03, 8.40)	0.606
ALC [10^9^/L, M (Q1, Q3)]	1.22 (0.82, 1.87)	1.20 (0.82, 1.84)	1.28 (0.82, 1.97)	0.389
AMC [10^9^/L, M (Q1, Q3)]	0.40 (0.31, 0.53)	0.40 (0.30, 0.52)	0.41 (0.32, 0.55)	0.262
Platelet [109/L, M (Q1, Q3)]	170.50 (123, 220)	170 (122.50, 219.50)	175 (124, 223.50)	0.794
Albumin [g/L, M (Q1, Q3)]	39.20 (36.40, 41.73)	39.20 (36.45, 41.70)	39.20 (36.35, 41.70)	0.966
SIRI [M (Q1, Q3)]	1.74 (0.96, 3.43)	1.95 (0.98, 3.39)	1.68 (0.93, 3.49)	0.646
NLR [M (Q1, Q3)]	4.85 (2.60, 7.63)	5.01 (2.67, 7.85)	4.27 (2.45, 7.35)	0.335
MLR [M (Q1, Q3)]	0.32 (0.22, 0.49)	0.32 (0.23, 0.48)	0.31 (0.21, 0.50)	0.750
PLR [M (Q1, Q3)]	135.02 (93.02, 191.51)	134.25 (96.40, 193.92)	139.13 (87.76, 185.47)	0.794
SAP, *n* (%)	129 (34.1)	96 (36.2)	33 (29.2)	0.187
**Clinical outcomes**				
Length of hospital stay, [days, M (Q1, Q3)]	15 (11, 20)	15 (11, 21)	15 (11, 19)	0.426
mRS score at 3 months [score, M (Q1, Q3)]	4 (2, 5)	4 (2, 5)	3 (2, 5)	0.575
Poor clinical outcome (mRS3–6) at 3 months, *n* (%)	220 (58.2)	152 (57.4)	68 (60.2)	0.611

SICH, spontaneous intracerebral hemorrhage; SAP, stroke-associated pneumonia; BMI, body mass index; NIHSS, National Institute of Health Stroke; Scale GCS, Glasgow Coma Scale; SBP, systolic blood pressure; DBP, diastolic blood pressure; IVH, intraventricular hemorrhage; WBC, white blood cell; ANC, absolute neutrophil count; ALC, absolute lymphocyte count; AMC, absolute monocyte count; SIRI, systemic inflammation response index; NLR, neutrophil/lymphocyte ratio; MLR, monocyte/lymphocyte ratio; PLR, platelet/lymphocyte ratio; M, mean or median; mRS, modified Rankin Scale.

# Statistically significant.

**Table 2:** General characteristics of patients with SICH according to the presence of SAP in the training cohort.

	**Total (*n* = 265)**	**SAP (*n* = 96, 36.2%)**	**Non-SAP (*n* = 169, 63.8%)**	***P***
**Demographics**				
Age [year, M (Q1, Q3)]	63.48 ± 13.75	67.2 ± 13.39	61.37 ± 13.55	0.001^#^
Gender, *n* (%)				0.871
Male	175 (66.0)	64 (66.7)	111 (65.7)	
Female	90 (34)	32 (33.3)	58 (34.3)	
BMI [kg/m^2^ M (Q1, Q3)]	24.49 (21.49, 27.04)	24.45 ± 4.41	24.60 ± 4.12	0.796
**Medical history**				
Hypertension, *n* (%)	198 (74.7)	79 (82.3)	119 (70.4)	0.032^#^
Diabetes mellitus, *n* (%)	20 (7.5)	10 (10.5)	10 (5.9)	0.183
Atrial fibrillation, *n* (%)	19 (7.2)	9 (9.4)	10 (5.9)	0.294
Smoking, *n* (%)	71 (26.8)	25 (26.0)	46 (27.2)	0.835
Drinking (>3 drinks per 24 h), *n* (%)	57 (21.5)	26 (27.1)	31 (18.3)	0.096
Antiplatelet or anticoagulation, *n* (%)	18 (6.8)	12 (12.5)	6 (3.6)	0.005^#^
**Clinical characteristics**				
NIHSS [score, M (Q1, Q3)]	3 (2, 7.5)	6 (3, 15)	3 (2, 6)	< 0.001^#^
GCS [score, M (Q1, Q3)]	13 (9, 15)	10 (7, 13)	14 (11, 15)	< 0.001^#^
Admission SBP [mmHg, M (Q1, Q3)]	164 (150, 181)	167 (150, 185)	162 (150, 180)	0.343
Admission DBP [mmHg, M (Q1, Q3)]	95.87 ± 16.12	92.50 (83.25, 103.75)	97 (86, 108)	0.099
Duration from onset to hospitalization [h, M (Q1, Q3)]	5 (3, 11)	5 (3, 11)	5 (3, 11.5)	0.389
Dysphagia, *n* (%)	123 (46.4)	71 (74)	52 (30.8)	< 0.001^#^
**ICH parameters**				
Hematoma volume[ml, M (Q1, Q3)]	12.54 (5.38, 23.02)	33.10 (18.17, 33.10)	8.93 (4.06, 17.17)	< 0.001^#^
Hematoma location, *n* (%)				0.156
Lobar	52 (19.6)	22 (22.9)	30 (17.8)	
Basal ganglia region	161 (60.8)	51 (53.1)	110 (65.1)	
Thalamus	52 (19.6)	23 (24.0)	29 (17.2)	
IVH, *n* (%)	79 (29.8)	43 (44.8)	36 (21.3)	< 0.001^#^
**Laboratory data**				
RBC [10^12^/L, (M ± SD)]	4.37 ± 0.62	4.27 ± 0.65	4.44 ± 0.60	0.030^#^
Hemoglobin [g/L, (M ± SD)]	135.03 ± 18.09	131.41 ± 18.53	137.10 ± 17.56	0.014^#^
WBC [10^9^/L, M (Q1, Q3)]	7.63 (6.27, 9.80)	9.06 (6.99, 11.82)	7.37 (5.98, 8.61)	< 0.001^#^
ANC [10^9^/L, M (Q1, Q3)]	5.53 (4.26, 8.03)	7.46 (4.97, 10.47)	5.15 (3.86, 6.57)	< 0.001^#^
ALC [10^9^/L, M (Q1, Q3)]	1.20 (0.82, 1.84)	0.90 (0.60, 1.35)	1.42 (0.99, 1.96)	< 0.001^#^
AMC [10^9^/L, M (Q1, Q3)]	0.40 (0.30, 0.52)	0.44 (0.28, 0.57)	0.39 (0.31, 0.51)	0.351
Platelet [10^9^/L, M (Q1, Q3)]	170 (122.50, 219.5)	159.50 (115, 219.75)	173 (130, 219)	0.254
Albumin [g/L, M (Q1, Q3)]	39.20 (34.45, 41.70)	38.40 (35.35, 41.8)	39.50 (37.1, 41.70)	0.068
SIRI [M (Q1, Q3)]	1.95 (0.98, 3.39)	3.04 (1.60, 5.19)	1.50 (0.87, 2.52)	< 0.001^#^
NLR [M (Q1, Q3)]	5.01 (2.67, 7.85)	7.09 (5.02, 12.39)	3.61 (2.33, 6.13)	< 0.001^#^
MLR [M (Q1, Q3)]	0.32 (0.23, 0.48)	0.44 (0.29, 0.63)	0.28 (0.21, 0.39)	< 0.001^#^
PLR [M (Q1, Q3)]	134.25 (96.40, 193.92)	154.51 (108.18, 267.32)	120.51 (86.67, 164.90)	< 0.001^#^
**Clinical outcomes**				
Length of hospital stay, [days, M (Q1, Q3)]	15 (11, 21)	19 (14, 16)	14 (11, 17)	< 0.001^#^
mRS score at 3 months [score, M (Q1, Q3)]	4 (2, 5)	5 (2, 5)	2 (2, 4)	< 0.001^#^
Poor clinical outcome (mRS3–6) at 3 months, *n* (%)	152 (57.4)	69 (71.9)	83 (49.1)	< 0.001^#^

SICH, spontaneous intracerebral hemorrhage; SAP, stroke-associated pneumonia; BMI, body mass index; NIHSS, National Institute of Health Stroke Scale; GCS, Glasgow Coma Scale; SBP, systolic blood pressure; DBP, diastolic blood pressure; IVH, intraventricular hemorrhage; WBC, white blood cell; ANC, absolute neutrophil count; ALC, absolute lymphocyte count; AMC, absolute monocyte count; SIRI, systemic inflammation response index; NLR, neutrophil/lymphocyte ratio; MLR, monocyte/lymphocyte ratio; PLR, platelet/lymphocyte ratio; M, mean or median; SD, standard deviation; mRS, modified Rankin Scale.

# Statistically significant.

### 3.2. Screening factors for SAP after SICH

The variables with *P* < 0.05 in the univariate analysis were included in the multivariate logistic regression analysis. IVH, hypertension, dysphagia, GCS, NIHSS, SIRI, and PLR were independent predictors of SAP after SICH (*P* < 0.05, Table 3). The Hosmer–Lemeshow test showed a good fit for the model (*P* = 0.961).

**Table 3:** Multivariate logistic regression analysis of the screening predictors of SAP after SICH.

**Variables**	**OR**	**95% CI**	***P***
IVH	2.909	1.384–6.113	0.005
Hypertension	2.810	1.174–6.722	0.020
Dysphagia	3.984	1.969–8.060	<0.001
GCS	0.885	0.783–1.000	0.049
NIHSS	1.133	1.053–1.218	0.001
SIRI	1.346	1.104–1.641	0.003
PLR	1.007	1.002–1.012	0.003

OR, odds ratio; CI, confidence interval; SICH, spontaneous intracerebral hemorrhage; SAP, stroke-associated pneumonia; NIHSS, National Institute of Health Stroke Scale; GCS, Glasgow Coma Scale; IVH, intraventricular hemorrhage; PLR, platelet/lymphocyte ratio.

### 3.3. A novel nomogram for SAP after SICH

**Figure 2:**
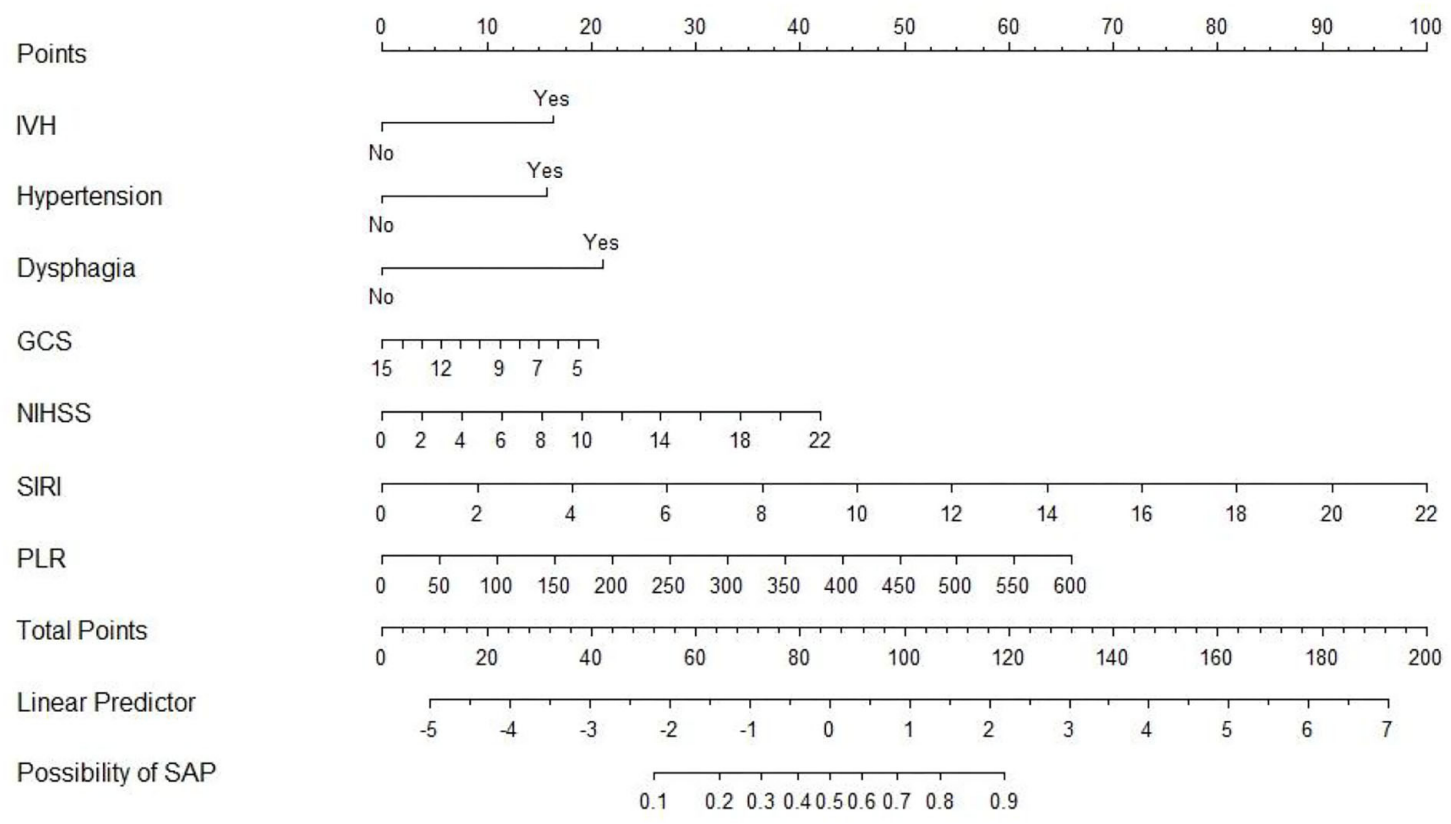
Nomogram for predicting SAP after SICH.

An SAP predictive nomogram was established using the seven significant predictors mentioned earlier (Figure 2). The predictors were scored, and then a straight line was plotted through the total score to investigate the likelihood of assessing post-SICH SAP based on the total score.

### 3.4. Predictive accuracy and net benefit of the nomogram

**Figure 3:**
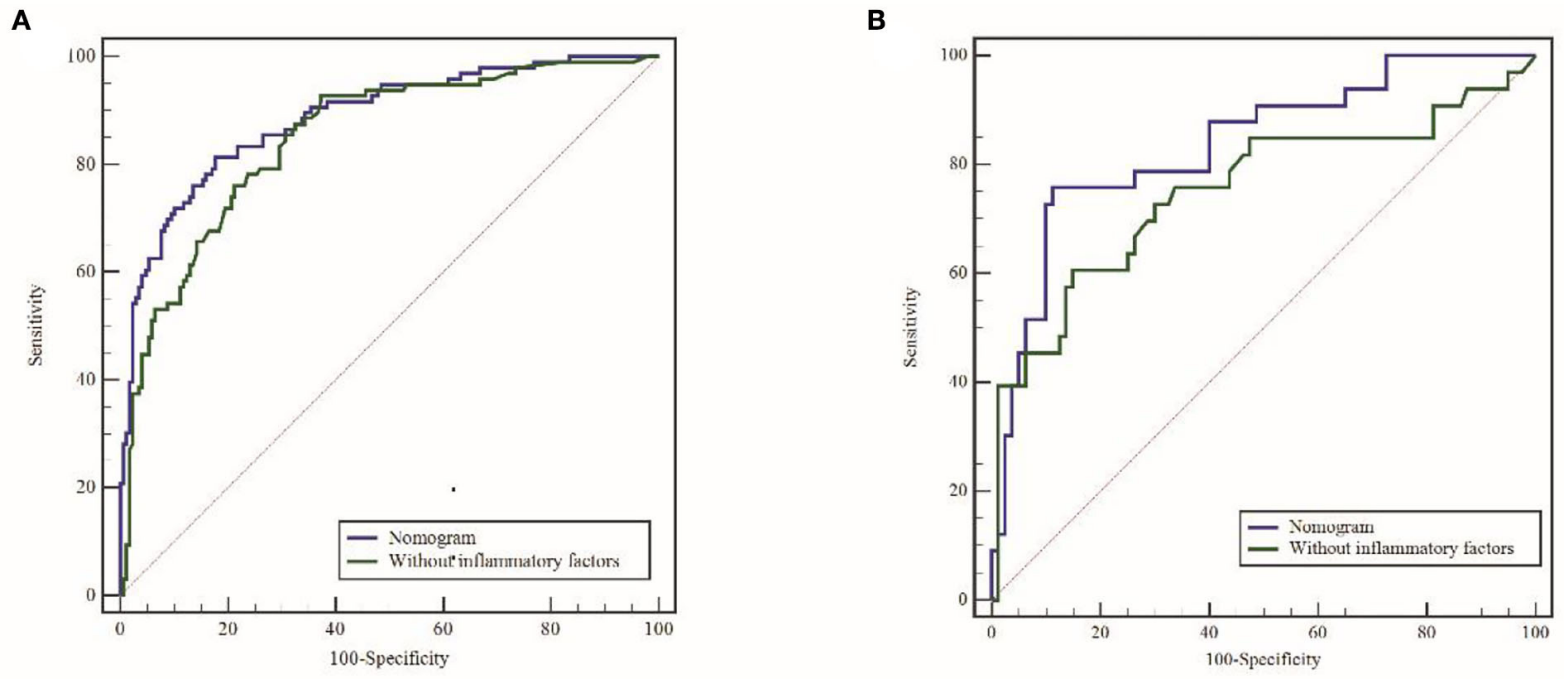
ROC curves for the raining cohort **(A)** and validation cohort **(B)**. ROC, receiver operating characteristic; AUC, area under the ROC.

**Figure 4:**
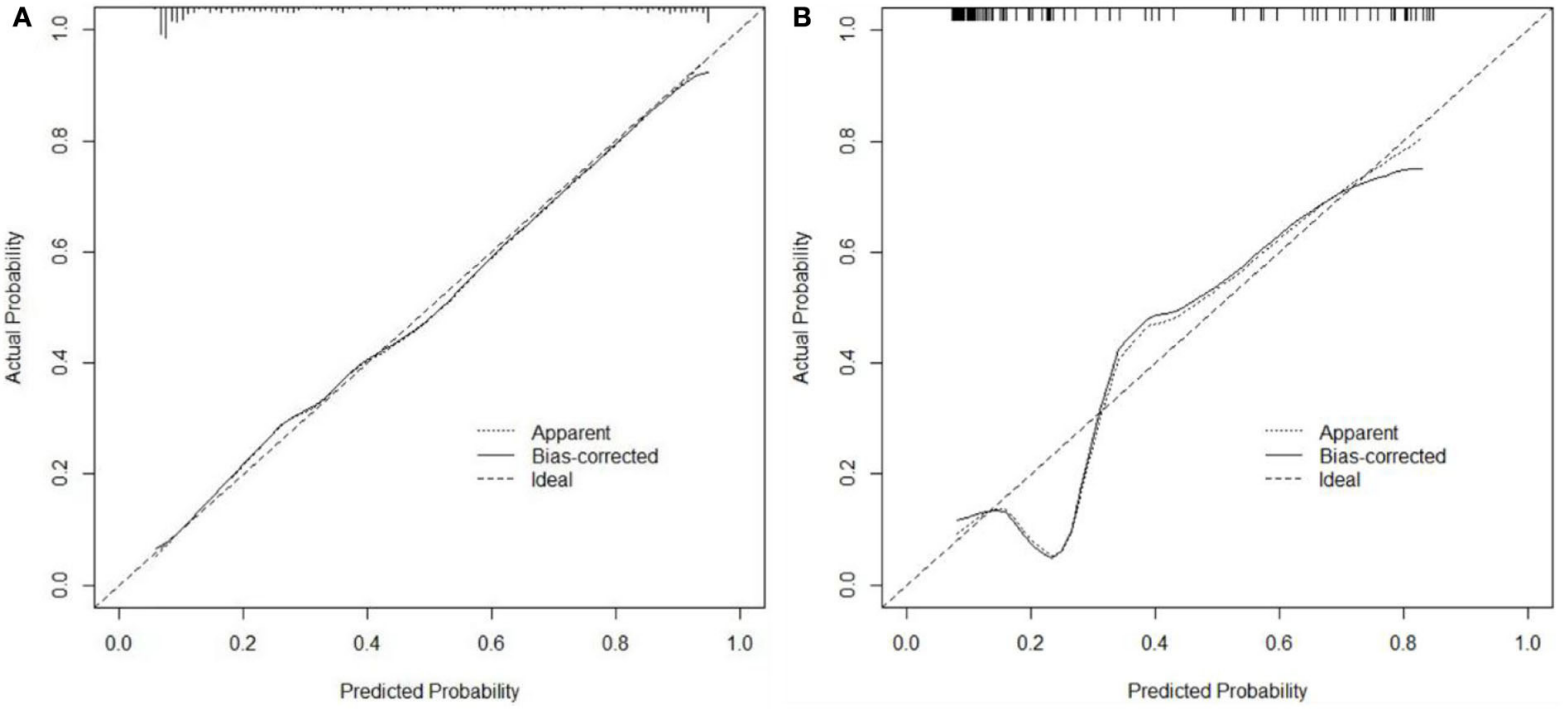
Calibration curves for predicting the probability of SAP after SICH. **(A)** Training cohort. **(B)** Validation cohort.

In the training cohort, ROC analysis revealed an AUC of 0.886 (95% CI: 0.841–0.921, *P* < 0.001) for the nomogram for SAP (Figure 3A), higher than that without inflammatory factors (SIRI and PLR) (AUC = 0.848, 95% CI: 0.799–0.899, *P* < 0.001). The calibration curve was close to the ideal diagonal (Figure 4A).

**Figure 5:**
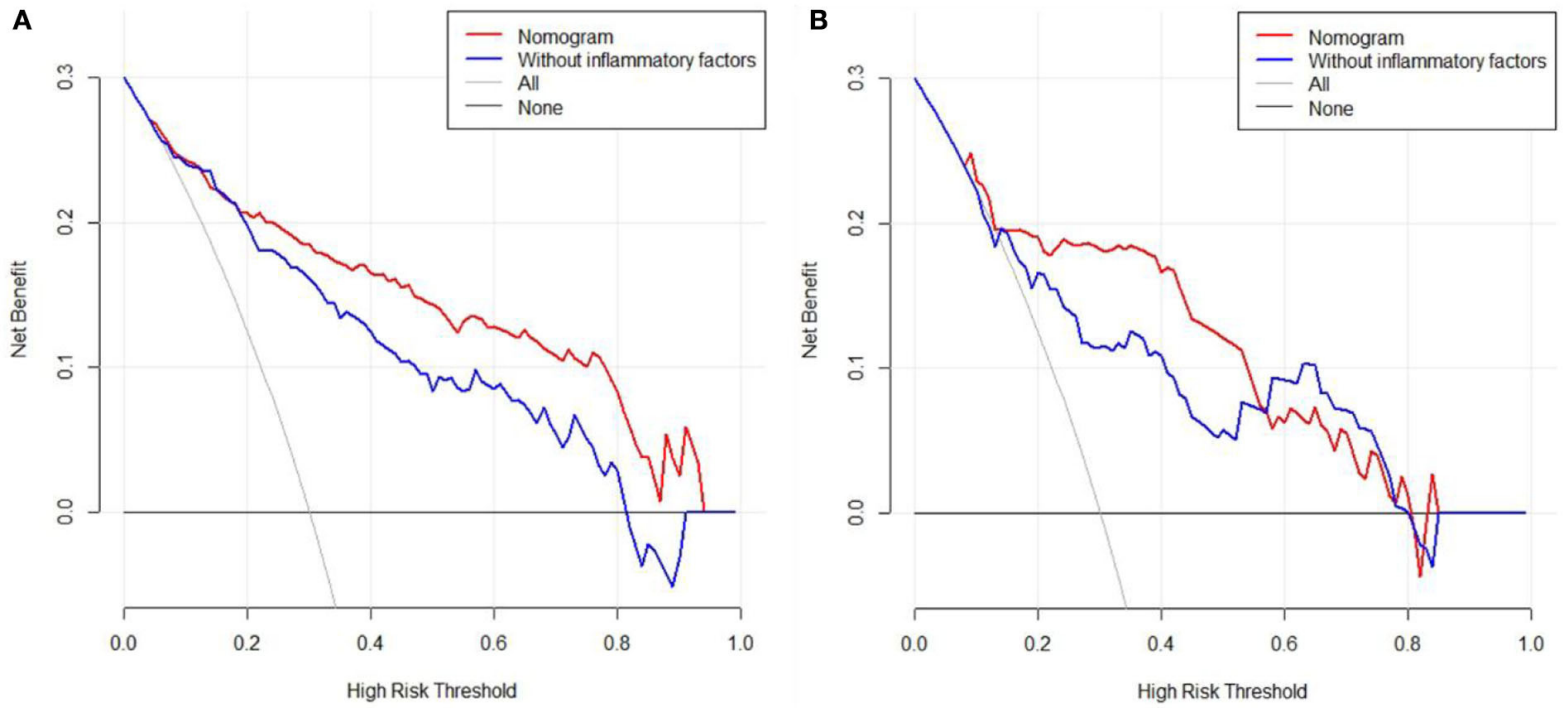
Decision curve analysis for the training cohort **(A)** and the validation cohort **(B)**.

Furthermore, 113 patients were utilized for the internal validation of the nomogram. The AUC of it was also 0.837 (95% CI: 0.756–0.900, *P* < 0.001), higher than the AUC without inflammatory factors (AUC = 0.752, 95% CI: 0.662–0.828, *P* < 0.001) (Figure 3B), confirming the nomogram's reliable accuracy. The calibration curve showed good consistency between the predicted and actual observed results in predicting SAP after SICH (Figure 4B). In addition, the DCA graph showed that the net benefit of the prediction model was better than that of the model without inflammatory factors over the risk range of SAP in both cohorts (Figure 5). These data suggest that our nomogram has important implications for clinical decision-making.

## 4. Discussion

Our single-center retrospective study showed that IVH, hypertension, dysphagia, GCS, NIHSS, SIRI, and PLR were independent predictors of SAP after SICH. We further developed a nomogram to predict the incidence of SAP after SICH by these seven essential predictors. This nomogram yielded better accuracy and presented better clinical utility for the individualized prediction of SAP after SICH compared with the conventional factors without inflammatory factors. Our study is the first to include inflammatory markers in a predictive model for predicting SAP after SICH. Furthermore, this study makes predicting the probability of SAP after SICH easier. In addition, the nomogram underwent rigorous internal validation, implying stable prognostic performance.

SAP is the most common stroke-associated infection that can prolong hospitalization and even severely influence the prognosis and mortality of stroke patients (5). Therefore, early determination of disease trends and aggressive and effective treatment and prevention of patients who may develop SAP can reduce adverse outcomes. Despite the clinical significance of SAP after SICH, no substantial progress has been made in preventing SAP, including the prophylactic use of antibiotics and the process of care (23). It is well known that inflammatory factors, namely NLR, PLR, MLR, and SIRI, are new composite inflammatory markers based on traditional inflammatory cell counts that provide a more comprehensive picture of the inflammatory symptom status of the body. Numerous clinical studies (14, 15, 24–27) have confirmed that the above indicators have good predictive value for the occurrence, development, and prognosis of tumors, stroke, and other diseases. However, do these indicators have a similar clinical value for SAP after SICH?

We analyzed the relationship between peripheral blood and SAP in patients with SICH on admission. This study showed that SICH patients with hypertension, IVH, dysphagia, higher NIHSS scores, and lower GCS scores were more likely to have SAP. These results are similar to previous studies (5–7). In addition, this study added some inflammatory markers according to inflammatory cells in peripheral blood. Patients with SICH with higher SIRI and PLR were more likely to develop SAP. This result may provide a new idea to differentiate SAP and non-SAP individuals after SICH quantitatively and to develop targeted medical interventions for individuals.

It was revealed that elevated SIRI is an independent indicator of poor prognosis in stroke (24–26), aneurysmal subarachnoid hemorrhage (28), and some tumors (29–31). Yan et al. (16) found that higher SIRI was a significant risk factor for pneumonia in patients with acute ischemic stroke. A SIRI threshold of ≥2.74 was correlated with an increased incidence of SAP in patients with AIS (OR: 5.82, 95% CI: 4.54, 7.49, *P* < 0.001). In addition, the RCS model showed an increasing trend in the risk of SAP with increasing SIRI. Our study included patients with SICH who received conservative treatment. These results were similar to the previous studies mentioned above. SIRI revealed a positive association with SAP after SICH.

It was reported that PLR is a prognostic indicator of inflammatory response in various conditions, such as acute pulmonary embolism (32), myocardial infarction (33), various cancers (34), and stroke (27). Deng et al. (35) revealed that PLR was a predictor of stroke-associated infection in patients with AIS. A recent study reported that changes in peripheral PLR during treatment could reflect disease progression and prognosis in patients with COVID-19. Furthermore, the greater ΔPLR correlated with a more severe cytokine storm, a longer hospital stay, and a worse prognosis (36). The predictive value of PLR in patients with SAP vs. SICH has not been investigated. Furthermore, the combined effect of inflammatory factors on SAP has been well reported. We built a new nomogram to predict SAP risk in patients with SICH during hospitalization. The nomogram we constructed with inflammatory factors showed better and more accurate predictions than the nomogram without inflammatory factors. Internal validation further validated the predictive ability of the nomogram. Therefore, SIRI and PLR should be considered when predicting SAP in patients with SICH receiving conservative treatment.

Consistent with other reports (16, 37), the hospital stay length was prolonged in the SAP group. Studies have shown that stroke-related infections, especially pneumonia, are independently associated with poor functional prognosis after stroke. Our study also observed that subjects with SAP had worse functional outcomes at 3-month follow-up, consistent with previous studies (38–40). Two phase-II studies on prophylactic antibiotic therapy showed benefits on temperature, the incidence of infection, and even functional outcomes (41, 42). The current management of SAP does not prescribe prophylactic antibiotics (43). The challenge now is to study the effect of preventive treatment on functional outcomes. A phase-III trial was conducted but was stopped early.

There may be several possible mechanisms between the inflammatory response and SAP. First, experimental studies have shown that many inflammatory processes occur after cerebral hemorrhage, including infiltration of leukocytes (44), activation of microglia (45), and release of inflammatory cytokines (46). However, brain injury affects the physiological interaction between the central nervous system and the immune system, resulting in a systemic immunosuppressive syndrome (47) manifested by a decrease in lymphocytes (40) that promotes susceptibility to infection. Finally, inflammatory factors may be the connecting point between cerebral hemorrhage severity and SAP.

Despite the good performance of our nomogram, several limitations should be noted in our study. First, incomplete statistical indicators, such as the time of SAP, pathogenic spectrum analysis, mechanical ventilation use, indwelling gastric tubes, and aspiration events, were not well documented. Therefore, this classification of suspected risk factors and pathogens was not included in the statistical analysis, resulting in the exclusion of confounding confounders in the multivariate logistic regression. Second, the retrospective analysis was limited to a single center, and did not further comparison of the dynamics of inflammatory indicators. Therefore, the results of this study were further validated in prospective multicenter cohort studies and other populations. Finally, the training and validation cohorts were from the same hospital. Therefore, multicenter studies need to seek external validation assessments before clinical application.

## 5. Conclusion

Admission SIRI and PLR can be utilized as potential prognostic inflammatory biomarkers in patients with SICH who underwent SAP. It helps to select high-risk patients for timely initiation of individualized therapy as these variables can be easily and rapidly obtained from blood cell counts. Combining nomograms for admission SIRI, PLR, and clinical risk factors will more reliably predict SAP in patients with SICH. In the future, large studies are needed to externally validate the nomograms for SAP after SICH in different populations. If proven valid, it will provide clinicians with an accurate and effective tool for early prediction and timely management of SAP after SICH. SAP can influence the length of hospital stay and the clinical outcome.

## Data availability statement

The raw data supporting the conclusions of this article will be made available by the authors, without undue reservation. Requests to access the datasets should be directed to JJ, jjx830829@163.com.

## Ethics statement

The studies involving human participants were reviewed and approved by the Ethics Committee of Taizhou People's Hospital. Written informed consent from the patients/participants or patients/participants' legal guardian/next of kin was not required to participate in this study in accordance with the national legislation and the institutional requirements.

## Author contributions

JJ and YL: study design, result interpretation, writing, reviewing, and editing. HL: data acquisition. TY: writing of the original draft and statistical analysis. All authors contributed to the execution of this work and the preparation of this manuscript. All authors have read and agreed to publish the final version of the manuscript.
